# Time to tighten the belts? Exploring the relationship between savings and obesity

**DOI:** 10.1371/journal.pone.0179921

**Published:** 2017-06-29

**Authors:** Karen Pickering, Mark Monahan, Alessandra Guariglia, Tracy E. Roberts

**Affiliations:** 1 Health Economics Unit, University of Birmingham, Birmingham, United Kingdom; 2 Department of Economics, University of Birmingham, Birmingham, United Kingdom; University of Exeter, UNITED KINGDOM

## Abstract

**Background:**

Literature suggests that the higher the rate of time preference people have, the less likely they are to save for the future. Likewise, it has been hypothesised that rising rates of being overweight/obesity are associated with an increase in peoples’ marginal rate of time preference.

**Aim:**

To investigate the relationship between being overweight/ obese and the rate of time preference in an older English population, using savings as a proxy for time preference.

**Methods:**

Three different econometric methods—Random-effects Probit Estimation, Fixed-effects Estimation, and Generalised Method of Moments Estimation—were used to explore the link between being overweight/ obese and rate of time preference in the English Longitudinal Study of Ageing dataset. Six waves of panel data spanning eleven years provided the data to test whether savings variables are related to being overweight/ obese.

**Results:**

The decision to save was shown to hold a statistically significant negative relationship with body mass index but only in the Generalised Method of Moments model. Placing savings in safe, low risk investments was significantly related to a lower probability of being obese but only in the random-effects Probit model. The proportion that people saved relative to their income was not found to be significantly associated with the probability of being overweight/ obese in any of the models.

**Conclusion:**

There is an unclear relationship between saving behaviour and being overweight/ obese in an older English population. A financial variable such as savings is a potentially appropriate but imperfect proxy for the rate of time preference of the population. Further research is required to clarify the relationship in order to help develop strategies for obesity prevention. The inconsistency in the results between methods highlights the importance of using a wide range of alternative techniques before implementing important policy decisions.

## Introduction

The prevalence of obesity in the UK is worrisome. Body mass index (BMI) [calculated as mass (kg)/height^2^ (m)] is used to assess whether individuals are overweight or obese. An individual is classified as overweight if they have a BMI value of 25 or over, and obese if they have a BMI value of 30 or over[1]. In 2014, 65% of all males and 58% of all females were classified as overweight, and around a quarter of all English males and females (24% and 27% respectively) were classified as obese[2]. Prevalence rates for the older population are of equal concern with around 80% of males and 70% of females aged 55 and over being classified as overweight, and around a third of males and females (30% and 33% respectively) being classified as obese[2]. Being overweight/ obese can cause complications related to diabetes and musculoskeletal issues[3]. The most up-to-date government report estimated the cost to the economy of obesity and being overweight to be £16 billion, with this figure predicted to rise to £50 billion if the situation is not controlled[4].

In order to combat an increasing and population wide obesity problem, it is important to understand the characteristics which lead people to become obese. Policymakers and practitioners can then target these characteristics to reduce obesity prevalence rates. The literature on the causes of obesity is extensive and so far several factors have been linked to the problem: food prices[5], socioeconomic status[6, 7], net worth[8] and debt[9, 10] to name a few. There is evidence to suggest that a person’s rate of time preference, which refers to the rate at which the individual discounts the future, may be a potential factor for explaining obesity levels[11–20]. People with a higher rate of time preference are more interested in present consumption at the expense of future consumption, compared to those with a lower rate, so it follows that any investments in health (e.g. exercise) where the benefits accrue in the future are less likely to be undertaken[15]. In contrast, if an individual considers saving to be a worthwhile activity, they are signalling that they are willing to forgo current consumption in favour of future consumption and therefore will hold a relatively low rate of time preference compared to those who do not save. Given this, ‘savings’ behaviour is generally considered an appropriate proxy to represent an individual’s rate of time preference[11, 15, 17].

The aim of this paper is to explore the relationship between being overweight/ obese and rate of time preference in an older UK population, using savings as a proxy for time preference.

## Methods

### Hypotheses

1. We assert a negative relationship between savings and being overweight/ obese. Those who have a high rate of time-preference choose to “live for today” and fail to save for the future. The same individuals are also more likely to put off exercising, and to indulge in food without thinking about the future consequences, thus, making them more likely to be overweight/ obese, compared to more patient individuals who save and value the future.
2. Additionally, we predict the relationship between being overweight/ obese and holding safe savings will be negative and more significant than the relationship between holding risky savings and being overweight/ obese. Those investing in safe forms of saving understand that they will definitely be able to access their savings in the future, whilst those investing in risky forms of savings may have a chance of losing their savings. We therefore predict that the overweight/ obese that do save will hold relatively more of their savings in risky forms, compared with safer forms of saving. The relationship between being overweight/ obese and holding safe savings will be stronger than the relationship between being overweight/ obese and holding risky savings. This is because those investing relatively more of their money in safe savings can be seen to value the future even more so.

### Data

We used data available in the English Longitudinal Study of Ageing (ELSA), a survey of people of 50 years of age or over and their partners living in the England. There are currently seven waves of data available, covering the years 2002 to 2015. Respondents complete the survey biannually. In waves 2, 4 and 6 (years 2004, 2008 and 2012 respectively), respondents were asked to comply to a nurse visit, where various health measures such as blood pressure, grip strength, height, weight and lung function were recorded[21].

### Sample

BMI values were recorded in waves 2, 4 and 6 only, so only these waves were used for analysis. Not all individuals were surveyed in all three waves of data, and so the panel was unbalanced.

Originally, the data sample, which included waves 2, 4 and 6, contained 33,960 observations. We identified partners younger than 50 years of age, observations without valid BMI values, observations without savings information and individuals residing in an institution, where personal control on diet and savings was likely to not exist. These were all excluded from our main sample so as not to skew the results. For transparency, descriptive statistics for those reporting BMI value were compared with those not reporting BMI value. We identified outliers (the top and bottom 1% of all observations) for the continuous variables and also excluded these as they can be considered extreme observations[22]. We also omitted observations with missing values for our regression variables. After these exclusions, 10,502 observations remained in our sample.

### Econometric methods

Our analysis used three alternative regression methods in parallel as there were three different dependent variables and each variable needed a suitable approach. We used random-effects Probit estimation to estimate the determinants of the probability of an individual being overweight or obese, and Fixed-effects (FE) and Generalised Method of Moments (GMM) models to estimate the determinants of BMI. All analyses were performed using the statistical software package Stata SE version 13 (StataCorp, Texas, USA).

Probit models are typically used to estimate the probability that the independent variable falls into one of two categories (zero or one). In our case, the Probit model was used to show the effect each characteristic has on the probability of an individual being overweight or obese. As panel data are used for our regression analysis, random-effects Probit models were used as they take into account the panel dimensions of the ELSA dataset, and enable the intercept to change randomly[23], controlling in this way for unobserved respondent-specific heterogeneity.

FE models take into account unobserved heterogeneity by allowing the intercept to change for each individual, remaining constant over time (i.e. across the waves of data[23]). The variables gender and ethnicity were omitted in the FE models through the differencing process, as these remain constant throughout all of the waves of data. One problem with both the random-effects Probit and the FE models is that they do not take into account the possible endogeneity of the regressors. So for example, if an independent variable (e.g. marital status) is affected by someone’s BMI value, then marital status is correlated with the error term in the model, meaning that both the random-effects Probit and FE estimates are biased and inconsistent.

GMM estimation uses information in population moment conditions (functions of unknown parameters) and combines this information with the observed data to produce parameter estimates[24]. The main advantage of the GMM estimator is that it controls for the possible endogeneity of the regressors, by using lags of these same regressors as instruments. As such, unlike FE estimation, GMM is able to account for the possibility that marital status (or any other independent variable) may be affected by BMI. GMM estimation also controls for unobserved respondent-specific heterogeneity.

In summary, all three models control for unobserved heterogeneity. Random-effects Probit models were used to allow the dependent variable to be dichotomous and to take into account the panel dimension of the dataset. FE is a commonly used technique for panel data, which has been used previously in similar studies with a continuous dependent variable[9, 10, 25]. GMM is a more sophisticated technique compared with FE as it controls for the possible endogeneity of the regressors.

### Variables

Dummy variables, indicating whether the individual is overweight [1] or not overweight [0], and obese [1] or not obese [0] were the dependent variables used for the random-effects Probit regression analysis. BMI value was used as the dependent variable for the FE and GMM regression analysis.

Independent variables were chosen in line with previous studies recognising the determinants of obesity[26, 27].Variables for age, annual income, gender, marital status, ethnicity, smoking, employment status, education, mobility and physical activity were included in all our models (Table 1).

**Table 1:** Variables for regression analysis.

Variable	Type	Units/Categories
BMI	Continuous	Body Mass Index value (*kg/m*^*2*^*)*
Overweight Dummy	Categorical	Overweight (if BMI 25 or over) [1]; Not Overweight (if BMI less than 25) [0]
Obesity Dummy	Categorical	Obese (if BMI 30 or over) [1]; Not Obese (if BMI less than 30) [0]
Age	Continuous	Years
Gender	Categorical	Male [1]; Female [0]
Ethnicity	Categorical	Non-White [1]; White [0];
Marital Status	Categorical	Married/Co-habiting [1]; Other [0]
Employment	Categorical	Working [1]; Not Working [0]
Education	Categorical	High (degree level or higher) [1]; Low [0]
Mobility	Categorical	Good (no problems with carrying out any of the surveyed daily activities) [1]; Bad (problems with at least one of the surveyed daily activities) [0]
Smoking	Categorical	Current smoker [1]; Not Current Smoker [0]
Income	Continuous	Log of the respondent’s income, where income is £ per year (sum of wages, state pensions, benefits and other income). Income is recorded in the previous 12 months before the survey is conducted
Physical Activity	Categorical	High (vigorous exercise at least once per week) [1]; Low [0]
Savings Dummy	Categorical	Save [1]; Do Not Save [0]
Savings Ratio	Continuous	Ratio between difference in total net financial wealth stock between waves over mean income between waves
Safe Savings Ratio	Continuous	Ratio between difference in total safe savings stock between waves over mean income between waves
Risky Savings Ratio	Continuous	Ratio between difference in total risky savings stock between waves over mean income between waves
Retired	Categorical	Retired [1]; Not Retired [0]

#### Savings

For the purpose of the current analysis, savings is defined as the difference in total net financial wealth between two periods. We created a savings ratio, showing the proportion of their income individuals save (Table 1). The savings ratio is calculated as the difference in total net financial wealth between two periods, divided by the mean income from the two periods[28]. Total net financial wealth and total income were deflated to real values using the retail price index (RPI). A dummy variable was also created to indicate whether or not respondents save between periods (Table 1).

The ELSA dataset provides information on the types of savings individuals hold and classifies them as “safe” and “risky”. Safe savings include bank accounts, savings accounts and cash individual savings accounts (ISAs), while risky savings include shares, bonds, stocks, shares ISAs or life insurance ISAs. Those investing more of their money in safe savings can be seen to value the future even more so, relative to those that choose to invest in risky savings.

Safe (risky) savings ratios were calculated as the difference in total safe (risky) savings between two periods, divided by the mean income from the two periods (Table 1).

### Analysis

Descriptive Statistics were identified, including two-sample t-tests, to test whether the differences between two variables are statistically significant. We tested whether savings is a valid proxy for time preference in our dataset by testing our savings variables against a “financial planning horizon” variable, available in wave 2 only. The financial planning horizon variable measures how far people plan ahead when spending or saving. We assumed a short planning horizon if the respondent did not plan or only planned for a few months ahead. A short planning horizon can be seen as a measure for time preference[29]. We therefore used this measure to check whether saving can indeed be seen as an alternative proxy for time preference. Specifically, we checked whether the differences in mean values of the savings dummy/ratio among people with and without a short planning horizon were statistically significant, using a t-test.

In order for the specification to comply with the model design, each form of regression analysis was estimated using the different measures of saving. Model 1 uses the savings dummy, model 2 the savings ratio, and model 3 uses the safe and risky savings ratios. For the random-effects Probit models, average marginal effects (AMEs) were calculated, to show the magnitude of the effect that the independent variables have on the dependent variable.

For each model we carried out appropriate goodness of fit tests. In all models (random-effects Probit, FE, and GMM), we report a test of joint significance of all the regressors. This is a Wald test in the case of the random effects Probit model and an F-test in the case of the FE and GMM models. For the random-effects Probit and FE models, we report the rho statistic, which denotes the proportion of the total variance contributed by the panel-level variance component. We also carried out the Hausman test (1978) to justify our use of a FE model rather than a random-effects model for BMI value. Finally, for the GMM models, relevant tests are the Sargan test and the test for second-order serial correlation of the residuals in the differenced equation (*m*2).

#### Secondary analyses

Further tests of significance were carried out following the main analysis. Given obesity prevalence levels increase with age and peak after retirement, in the 65–74 age bracket[30], a “retired” variable was also created for a secondary analysis to explore the potential effect of retirement on the likelihood of being overweight /obese. Thus, all regressions were repeated replacing “employment” with “retired”, a new dummy variable to indicate whether the individual is retired or not.

A number of interaction tests were also carried out on significant savings variables to check for any differences between subgroups.

We split the sample at the mean age (69 years) to check for any differences between the older and younger subgroups, as we acknowledge that the two subgroups may have different rates of time preference due to their different life expectancy. We predict the younger subgroup to show a stronger negative relationship between savings and overweight/obesity, as they should be relatively more forward thinking in terms of planning ahead, relative to the older population who have predominantly less time left to live.

## Results

### Descriptive statistics

Summary statistics for the study population are presented in Table 2. The sample comprised 45% men and 55% women, the mean age was 66 years and the mean BMI was 28.1. 72% of the sample were classified as overweight and 31% of the sample were obese which is in line with other UK statistics[2]. Looking at the split of the savings dummy, half of the sample were shown to save between waves (50.33% save versus 49.67% that do not save). The only highly correlated variables (correlation >0.5) were age and retired (see S1 Table). The summary statistics for observations reporting BMI value and observations not reporting BMI value were compared (Table 3). Significant differences were found between the two groups for variables gender, ethnicity, education, mobility, smoking, income, physical activity and retired. No significant differences were found in the savings variables.

**Table 2:** Summary statistics.

Variable	Mean	Std. Dev.	Min	Max
BMI value	28.131	4.695	18.656	44.296
Overweight Dummy	0.730	0.444	0.000	1.000
Obesity Dummy	0.310	0.462	0.000	1.000
Age	69.021	8.749	54.000	99.000
Gender	0.446	0.497	0.000	1.000
Ethnicity	0.021	0.144	0.000	1.000
Marital Status	0.690	0.463	0.000	1.000
Employment	0.267	0.442	0.000	1.000
Education	0.162	0.368	0.000	1.000
Mobility	0.434	0.496	0.000	1.000
Smoking	0.105	0.307	0.000	1.000
Income	9.639	0.548	7.584	11.125
Physical Activity	0.294	0.456	0.000	1.000
Savings Dummy	0.503	0.500	0.000	1.000
Savings Ratio	-0.007	3.085	-15.318	15.599
Safe Savings Ratio	0.025	1.903	-10.021	9.304
Risky Savings Ratio	-0.052	1.944	-10.864	11.490
Retired	0.647	0.478	0.000	1.000

Summary statistics based on 10,502 observations

**Table 3:** Summary statistics for those not reporting BMI value.

Variable	Mean	Std. Dev.	Min	Max	Diff ^1^
Age	66.395	11.743	50	99	0.511
Gender	0.436	0.496	0	1	0.003*
Ethnicity	0.051	0.219	0	1	0.000*
Marital Status	0.700	0.458	0	1	0.046
Employment	0.367	0.482	0	1	0.038
Education	0.145	0.352	0	1	0.004*
Mobility	0.411	0.492	0	1	0.000*
Smoking	0.171	0.377	0	1	0.000*
Income	9.520	0.612	7.418	11.148	0.000*
Physical Activity	0.240	0.427	0	1	0.000*
Savings Dummy	0.466	0.499	0	1	0.000*
Savings Ratio	0.055	2.999	-15.118	15.388	0.522
Safe Savings Ratio	0.526	0.499	0	1	0.384
Risky Savings Ratio	0.040	1.891	-9.701	9.614	0.861
Retired	-0.028	1.834	-10.968	11.481	0.785

Summary statistics based on 1,420 observations

^1.^ This column reports the p-value for the t-test for whether the difference in the mean of each variable is statistically different across the 2 groups (BMI value reported vs BMI value not reported).

* indicates that the difference between the 2 groups is statistically significant (P-value<0.005).

The results of the two sample t-tests, showing the difference in the savings ratio between those classified as overweight and those that are not overweight, was found to be negatively significant at the 10% level (Table 4). No significant differences were found when looking at the savings ratio with regards to obesity, or when looking at the safe and risky savings ratios.

**Table 4:** Two sample T tests.

	Savings Ratio t value	Safe Savings Ratio t value	Risky Savings Ratio t value
**Overweight**	-1.773*	-1.283	-0.729
**Obese**	-0.914	0.696	-0.414

Figures in this Table represent test statistics for whether the difference in savings ratios between those that are overweight/obese and those that are not overweight/obese are statistically significant.

*indicates statistical significance at the 10% level;

** at the 5% level;

*** at the 1% level.

The mean value of the savings dummy was found to be lower for respondents with a short planning horizon relative to those without (0.485 vs 0.526). The difference between the two dummies was statistically significant at the 1% level. Similarly, the mean value of the savings ratio was found to be lower for respondents with a short planning horizon compared to those without (-0.000 vs 0.003). The difference between the two ratios was statistically significant at the 10% level.

### Regression results

Results from the three different model specifications on savings and obesity are shown in Tables 5, 6, 7 and 8. Table 5 presents the results of the random-effects Probit models for the probability of being overweight and Table 6 shows the results of the random-effects Probit models for the probability of being obese. Table 7 provides the results of the FE models and Table 8 the results of the GMM models for BMI value.

**Table 5:** Random-effects Probit model for the probability of being overweight.

Variable	Model 1: Savings Dummy	Model 2: Savings Ratio	Model 3: Safe and Risky Savings Ratios
Overweight Dummy Variable	Coefficient (Standard errors in parentheses; Average Marginal Effects in square brackets)	Coefficient (Standard errors in parentheses; Average Marginal Effects in square brackets)	Coefficient (Standard errors in parentheses; Average Marginal Effects in square brackets)
Age	-0.050***	-0.050***	-0.052***
	(0.007)	(0.007)	(0.007)
	[-0.002]	[-0.003]	[-0.003]
Gender	1.126***	1.107***	1.116***
	(0.109)	(0.108)	(0.109)
	[0.047]	[0.058]	[0.056]
Ethnicity	0.244	0.282	0.378
	(0.339)	(0.337)	(0.342)
	[0.010]	[0.015]	[0.019]
Marital Status	0.482***	0.464***	0.458***
	(0.116)	(0.115)	(0.116)
	[0.020]	[0.024]	[0.023]
Employment	-0.254**	-0.263***	-0.292**
	(0.117)	(0.115)	(0.118)
	[-0.011]	[-0.014]	[-0.015]
Education	-0.899***	-0.862***	-0.894***
	(0.149)	(0.148)	(0.150)
	[-0.038]	[-0.045]	[-0.045]
Mobility	-1.030***	-1.021***	-1.031***
	(0.094)	(0.093)	(0.094)
	[-0.043]	[-0.053]	[-0.052]
Smoking	-1.957***	-1.890***	-1.919***
	(0.185)	(0.178)	(0.180)
	[-0.082]	[-0.098]	[-0.097]
Income	0.039	0.022	0.058
	(0.087)	(0.087)	(0.088)
	[0.002]	[0.001]	[0.003]
Physical Activity	-0.481***	-0.490***	-0.483***
	(0.094)	(0.094)	(0.095)
	[-0.020]	[-0.026]	[-0.024]
Savings Ratio	−	0.014	−
		(0.011)	
		[0.001]	
Savings Dummy	-0.061	−	−
	(0.071)		
	[-0.003]		
Safe Savings Ratio	−	−	-0.003
			(0.018)
			[-0.000]
Risky Savings Ratio	−	−	0.024
			(0.019)
			[0.001]
Intercept	6.112***	5.989***	5.829***
	(0.949)	(0.943)	(0.955)
Rho	0.944	0.937	0.938
Wald Test	370.13	378.45	375.48
Degrees of freedom	11	11	12
p-value	0.000	0.000	0.000

*indicates statistically significant at the 10% level;

** at the 5% level;

*** at the 1% level.

**Table 6:** Random-effects Probit model for the probability of being obese.

Variable	Model 1: Savings Dummy	Model 2: Savings Ratio	Model 3: Safe and Risky Savings Ratios
Obese Dummy Variable	Coefficient (Standard errors in parentheses; Average Marginal Effects in square brackets)	Coefficient (Standard errors in parentheses; Average Marginal Effects in square brackets)	Coefficient (Standard errors in parentheses; Average Marginal Effects in square brackets)
Age	-0.082***	-0.084***	-0.085***
	(0.007)	(0.007)	(0.007)
	[-0.008]	[-0.008]	[-0.008]
Gender	-0.116	-0.103	-0.130
	(0.110)	(0.111)	(0.113)
	[-0.011]	[-0.010]	[-0.012]
Ethnicity	0.212	0.216	0.287
	(0.371)	(0.375)	(0.382)
	[0.020]	[0.020]	[0.027]
Marital Status	-0.052	-0.039	-0.032
	(0.117)	(0.119)	(0.120)
	[-0.005]	[-0.004]	[-0.003]
Employment	-0.109	-0.150	-0.160
	(0.118)	(0.120)	(0.121)
	[-0.010]	[-0.014]	[-0.015]
Education	-0.791***	-0.802***	-0.827***
	(0.144)	(0.146)	(0.148)
	[-0.074]	[-0.076]	[-0.078]
Mobility	-1.437***	-1.455***	-1.450***
	(0.098)	(0.099)	(0.101)
	[-0.135]	[-0.137]	[-0.137]
Smoking	-1.248***	-1.294***	-1.311***
	(0.161)	(0.164)	(0.166)
	[-0.117]	[-0.122]	[-0.124]
Income	-0.248***	-0.279***	-0.242***
	(0.087)	(0.089)	(0.091)
	[-0.023]	[-0.026]	[-0.023]
Physical Activity	-0.728***	-0.741***	-0.743***
	(0.098)	(0.099)	(0.100)
	[-0.068]	[-0.070]	[-0.070]
Savings Ratio	−	-0.008	−
		(0.011)	
		[-0.001]	
Savings Dummy	-0.054	−	−
	(0.071)		
	[-0.005]		
Safe Savings Ratio	−	−	-0.054***
			(0.019)
			[-0.005]
Risky Savings Ratio	−	−	0.011
			(0.019)
			[0.001]
Intercept	7.105***	7.510***	7.251***
	(0.964)	(0987)	(1.000)
Rho	0.935	0.937	0.937
Wald Test	432.66	431.78	424.71
Degrees of freedom	11	11	12
p-value	0.000	0.000	0.000

*indicates statistically significant at the 10% level;

** at the 5% level;

*** at the 1% level.

**Table 7:** Fixed effects models.

Variable	Model 1: Savings Dummy	Model 2: Savings Ratio	Model 3: Safe and Risky Savings Ratios
BMI value	Coefficient (Standard errors in parentheses)	Coefficient (Standard errors in parentheses)	Coefficient (Standard errors in parentheses)
Age	-0.021	-0.013	-0.013
	(0.039)	(0.040)	(0.040)
Gender	0.000	0.000	0.000
	(omitted)	(omitted)	(omitted)
Ethnicity	0.000	0.000	0.000
	(omitted)	(omitted)	(omitted)
Marital Status	0.230	0.311*	0.320*
	(0.164)	(0.167)	(0.169)
Employment	-0.103	-0.114	-0.150
	(0.089)	(0.090)	(0.092)
Education	0.084	0.048	0.072
	(0.291)	(0.299)	(0.306)
Mobility	-0.206***	-0.192***	-0.175**
	(0.070)	(0.070)	(0.072)
Smoking	-1.480***	-1.449***	-1.477***
	(0.183)	(0.187)	(0.192)
Income	-0.058	-0.072	-0.054
	(0.067)	(0.069)	(0.071)
Physical Activity	-0.048	-0.050	-0.053
	(0.066)	(0.067)	(0.069)
Savings Ratio	−	0.003	−
		(0.007)	
Savings Dummy	0.001	−	−
	(0.042)		
Safe Savings Ratio	−	−	-0.003
			(0.011)
Risky Savings Ratio	−	−	0.006
			(0.012)
Intercept	30.259***	29.773***	29.622***
	(2.843)	(2.885)	(2.923)
Rho	0.929	0.929	0.928
F-test	8.28	7.72	6.90
Degrees of freedom	10	10	11
p-value	(0.000)	(0.000)	(0.000)
Hausman Test	351.85	353.55	347.29
(p-value)	(0.000)	(0.000)	(0.000)

*indicates statistically significant at the 10% level;

** at the 5% level;

*** at the 1% level.

**Table 8:** GMM models.

Variable	Model 1: Savings Dummy	Model 2: Savings Ratio	Model 3: Safe and Risky Savings Ratios
BMI value	Coefficient (Standard errors in parentheses)	Coefficient (Standard errors in parentheses)	Coefficient (Standard errors in parentheses)
Age	-0.219***	-0.253*	-1.267
	(0.072)	(0.149)	(12.784)
Gender	0.900**	1.012	6.541
	(0.374)	(0.692)	(73.474)
Ethnicity	-0.738	-1.393	-17.445
	(1.166)	(2.054)	(199.791)
Marital Status	0.667	-0.032	10.401
	(1.003)	(1.445)	(107.791)
Employment	0.842	-0.729	3.552
	(1.330)	(1.930)	(22.919)
Education	0.365	-0.733	14.986
	(1.685)	(2.296)	(165.899)
Mobility	-0.981	-1.240	11.271
	(0.707)	(1.016)	(164.753)
Smoking	-4.126	-8.483	-0.455
	(3.764)	(7.523)	(151.690)
Income	-1.512	1.618	-10.076
	(4.115)	(6.415)	(64.948)
Physical Activity	-9.705*	-9.056	-112.603
	(5.675)	(9.098)	(1342.164)
Savings Ratio	−	-3.347	−
		(3.168)	
Savings Dummy	-12.736*	−	−
	(7.027)		
Safe Savings Ratio	−	−	-25.417
			(226.933)
Risky Savings Ratio	−	−	-25.513
			(355.481)
Intercept	67.006	33.877	228.854
	(41.923)	(58.514)	(1347.462)
F-statistic	9.67	4.67	0.09
Degrees of freedom	13	13	14
P-value	0.000	0.000	1.000
Sargan Test	0.467	0.432	Not reported^1^

*indicates statistically significant at the 10% level;

** at the 5% level;

*** at the 1% level.

^1.^ Sargan test is not reported as The two-step estimated covariance matrix of moments is singular.

#### Random-effects Probit models for the probability of being overweight

From the random-effects Probit models for the probability of being overweight (Table 5), the coefficients associated with the savings ratio, savings dummy, safe and risky savings are not shown to be statistically significant. Those that are married/cohabiting and males have a higher and statistically significant (α = 0.05) probability of being overweight. Conversely, age, employment, education level, mobility and smoking status are negatively and significantly associated (α = 0.05) with the probability of being overweight. For example, those that are married /cohabiting show a 2% higher probability of being overweight, while a high education level is associated with a 4%-5% lower probability of being overweight.

#### Random-effects Probit models for the probability of being obese

The coefficients associated with the savings ratio, savings dummy and risky savings ratio are not found to be statistically significant in the random-effects Probit models (Table 6). The coefficient on the safe savings ratio is found to be negative and statistically significant at the 1% level. AMEs show a 10% increase in an individual’s safe savings ratio is associated with a 0.05 percentage point lower probability of being obese.

Statistically significant coefficients are also shown for the variables of age, education, smoking, mobility level, physical activity and income (see Table 6).

- A one year increase in age is shown to be associated with an 8.4 percentage point lower probability of being obese.
- A high education level is associated with a 7%-8% lower probability of being obese.
- Smoking is associated with a 12% lower probability of being obese.
- A good mobility level is associated with a 14% lower probability of being obese and high levels of physical activity, with a 7% lower probability.
- A 10% increase in income is associated with a 0.26 percentage point lower probability of being obese.

#### Fixed-effects

Table 6 presents the results of the Fixed-effects models, explaining BMI. BMI is found to be negatively correlated with mobility level and smoking (both at 1% level), and positively correlated with marital status (10% level). None of the savings variables were shown to be statistically significant in predicting a person’s BMI value.

#### Generalised Method of Moments

Table 8 presents the results of the GMM models aimed at explaining BMI. The savings dummy is found to be negatively significantly correlated with BMI value at the 10% level of significance. The coefficients associated with the savings ratio, safe savings ratio and risky savings ratio are insignificant.

Age is found to be negatively correlated with BMI value in both the models with the savings ratio and the savings dummy, at the 10% and 1% levels of significance respectively. In Model 1 only, gender was found to be positively correlated (5%), and physical activity negatively correlated (10% level) with BMI value.

#### Goodness of fit

Goodness of fit tests for each model are reported at the bottom of the results tables (Tables 5–8). For the tests of joint significance of all the regressors, in most cases, we obtain a p-value equal to 0.00, which suggests that all our regressors are jointly significant. For the random-effects Probit models and fixed effects models, we can see that rho takes on very high values, which justifies our use of these models. The Hausman test leads to a strong rejection of the null hypothesis that the random-effects model provides consistent estimates, which justifies our use of fixed effects estimation for BMI value. For GMM estimation, we were unable to obtain the m2 test as our dataset only contained three years of observations. However, the Sargan test enables us to accept the null hypothesis that the model is correctly specified.

### Secondary analyses

#### Retired

All regressions and models were run again replacing “employment” with “retired.” In the random-effects Probit estimation for the probability of being overweight models 1, 2 and 3 all showed being retired to be positively and significantly related to an individual’s probability of being overweight, with AMEs showing being retired is associated with a 1% higher probability of being overweight (S2 Table). Being retired was not shown to be significant in any of the other regressions (random-effects Probit estimation for the probability of being obese, FE, GMM) (see S3, S4 and S5 Tables).

#### Interactions

Differences between subgroups of the population were investigated for the significant savings results found from earlier analysis. The significant savings results were the safe savings ratio (Model 3) in the random-effects Probit regression for the probability of being obese, and the savings dummy (Model 1) in the GMM estimation.

#### Random-effects Probit regression for the probability of being obese: Model 3

Table 9 presents a comparison of the coefficients associated with the safe savings ratios in the random-effects Probit regression for the probability of being obese across different groups of the population. The coefficient on the safe savings variable is found to be significant for both males and females, but larger for males. A 10% increase in the safe savings ratio is related with a 0.06 percentage point lower probability of being obese for males, and a 0.05 percentage point lower probability for women. The difference between the two coefficients is not statistically significant.

**Table 9:** Further regression analysis: Safe savings ratio (Random-effects Probit model for the probability of being obese).

	Coefficient (Standard errors in parentheses; Average Marginal Effects in square brackets)		Chi Squared
**Gender**	*Males*	*Females*	
*Safe Savings Ratio*	-0.059**	-0.049*	0.06
	(0.028)	(0.260)	
	[-0.006]	[-0.005]	
**Ethnicity**	*Non-white*	*White*	
*Safe Savings Ratio*	-0.394**	-0.049**	2.95*
	(0.200)	(0.0193)	
	[-0.037]	[-0.005]	
**Marital Status**	*Married/co-habiting*	*Not Married*	
*Safe Savings Ratio*	-0.595***	-0.035	0.30
	(0.221)	(0.038)	
	[-0.006]	[-0.003]	
**Employment**	*Working*	*Not Working*	
*Safe Savings Ratio*	-0.053	-0.054**	0.00
	(0.046)	(0.021)	
	[-0.005]	[-0.005]	
**Education**	*High*	*Low*	
*Safe Savings Ratio*	0.034	-0.076***	5.19**
	(0.431)	(0.022)	
	[0.003]	[-0.007]	
**Mobility**	*Good*	*Bad*	
*Safe Savings Ratio*	-0.034	-0.068***	0.75
	(0.029)	(0.026)	
	[-0.003]	[-0.006]	
**Smoking**	*Current Smoker*	*Not Current Smoker*	
*Safe Savings Ratio*	-0.044	-0.054***	0.02
	(0.079)	(0.020)	
	[-0.004]	[-0.005]	
**Physical Activity**	*High*	*Low*	
*Safe Savings Ratio*	-0.046	-0.057**	0.06
	(0.037)	(0.023)	
	[-0.004]	[-0.005]	
**Retired**	*Retired*	*Not Retired*	
*Safe Savings Ratio*	-0.044*	-0.080**	0.61
	(0.023)	(0.039)	
	[-0.004]	[-0.008]	
**Age**	*65 or over*	*Less than 65*	
*Safe Savings Ratio*	-0.044*	-0.076**	0.56
	(0.023)	(0.035)	
	[-0.004]	[-0.007]	
**Age**	*80 or over*	*Less than 80*	
*Safe Savings Ratio*	-0.011	-0.061***	0.83
	(0.051)	(0.021)	
	[-0.001]	[-0.006]	
**Age**	*90 or over*	*Less than 90*	
*Safe Savings Ratio*	0.011	-0.054***	0.07
	(0.252)	(0.019)	
	[0.001]	[-0.005]	

*indicates statistically significant at the 10% level;

** at the 5% level;

*** at the 1% level.

The coefficient on the safe savings variable is also significant both for white and non-white people, with the difference between the coefficients being statistically significant. A 10% increase in the safe savings ratio is related with a 0.37 percentage point lower probability of being obese for those who are non-white, but only a 0.05 percentage point lower for those who are white.

The coefficient on safe savings is negatively and significantly associated with the probability of being obese for those that have a low education level, but not significant for those with a high education level. The difference between the coefficients of the two groups is statistically significant.

Safe savings is also negatively and significantly related with an individual’s probability of being obese for those that are married or co-habiting, have bad mobility and are not current smokers at the 1% level of significance, and for those that are not working and have a low level of physical activity at the 5% level of significance. The same holds for both retired and non-retired individuals. A 10% increase in the safe savings ratio is related with a 0.04 percentage point lower probability of being obese for those that are retired, and a 0.08 percentage point lower probability for those that are not retired. The difference between the coefficients is not statistically significant.

When comparing those that are 65 or over and those that are younger than 65, both groups’ safe savings ratios are negatively and significantly related to the probability of being obese. However when splitting the sample at age 80 and age 90, only those younger than 80 or 90 show a negative association between safe savings ratios and the probability of being obese, at the 1% level of significance.

#### GMM: Model 1

The subgroup results of the GMM model showed those with a low education level to have a negative and significant coefficient on the savings dummy (S6 Table). Coefficients for those with a high level of education were not statistically significant. No other coefficients on the savings dummy were found to be statistically significant. No statistically significant differences were found between subgroup coefficients.

#### Subgroups by age

When splitting the sample into younger (aged 50–69) and older (aged 70+) subgroups, there are some significant differences between the subgroups. The coefficient associated with the savings dummy is negatively and significantly associated with the probability of being overweight for the younger respondents only (Table 10). The coefficient associated with the risky savings ratio is positively and significantly associated with the probability of being overweight for the older subgroup only (Table 10). The coefficient associated with the safe savings ratio is negatively and significantly associated with the probability of being obese for both subgroups, as it was in the main analysis (S7 Table). For the FE models, as in the main analysis, none of the savings variables are shown to be statistically significant in predicting a person’s BMI value (S8 Table). Similarly, the GMM models show the savings dummy to no longer be statistically significant in either of the subgroups (S9 Table).

**Table 10:** Random-effects Probit model for the probability of being overweight—Split sample by age.

Variable	Model 1: Savings Dummy		Model 2: Savings Ratio		Model 3: Safe and Risky Savings Ratios	
Overweight Dummy Variable	Coefficient (Standard errors in parentheses; Average Marginal Effects in square brackets)		Coefficient (Standard errors in parentheses; Average Marginal Effects in square brackets)		Coefficient (Standard errors in parentheses; Average Marginal Effects in square brackets)	
	Aged 50–69	Aged 70+	Aged 50–69	Aged 70+	Aged 50–69	Aged 70+
Age	0.021	-0.111***	0.022	-0.099***	0.017	-0.102***
	(0.017)	(0.013)	(0.018)	(0.011)	(0.018)	(0.012)
	[0.001]	[-0.007]	[0.001]	[-0.009]	[0.001]	[-0.009]
Gender	1.389***	0.897***	1.366***	0.808***	1.390***	0.813***
	(0.149)	(0.163)	(0.153)	(0.151)	(0.155)	(0.154)
	[0.043]	[0.055]	[0.045]	[0.076]	[0.042]	[0.073]
Ethnicity	0.631	-0.302	0.668	-0.282	0.839*	-0.328
	(0.424)	(0.587)	(0.431)	(0.539)	(0.444)	(0.553)
	[0.020]	[-0.019]	[0.022]	[-0.027]	[0.025]	[-0.030]
Marital Status	0.442***	0.431***	0.410**	0.359**	0.379**	0.389**
	(0.169)	(0.160)	(0.173)	(0.148)	(0.174)	(0.151)
	[0.014]	[0.026]	[0.014]	[0.034]	[0.011]	[0.035]
Employment	0.113	-0.267	0.087	-0.207	0.024	-0.155
	(0.140)	(0.320)	(0.142)	(0.297)	(0.145)	(0.305)
	[0.004]	[-0.016]	[0.003]	[-0.020]	[0.001]	[-0.014]
Education	-0.865***	-0.892***	-0.840***	-0.768***	-0.861***	-0.818***
	(0.185)	(0.253)	(0.191)	(0.227)	(0.193)	(0.235)
	[-0.027]	[-0.055]	[-0.028]	[-0.072]	[-0.026]	[-0.074]
Mobility	-1.330***	-0.913***	-1.327***	-0.876***	-1.326***	-0.906***
	(0.133)	(0.140)	(0.135)	(0.129)	(0.138)	(0.133)
	[-0.041]	[-0.056]	[-0.044]	[-0.083]	[-0.040]	[-0.082]
Smoking	-1.859***	-2.528***	-1.845***	-2.118***	-1.873***	-2.182***
	(0.222)	(0.338)	(0.230)	(0.270)	(0.233)	(0.280)
	[-0.058]	[-0.155]	[-0.061]	[-0.200]	[-0.056]	[-0.197]
Income	0.017	0.006	0.003	-0.007	0.070	-0.003
	(0.116)	(0.141)	(0.117)	(0.131)	(0.119)	(0.134)
	[0.001]	[0.000]	[0.000]	[-0.001]	[0.002]	[0.000]
Physical Activity	-0.706***	-0.354**	-0.724***	-0.362**	-0.707***	-0.363**
	(0.126)	(0.153)	(0.128)	(0.144)	(0.130)	(0.147)
	[-0.022]	[-0.022]	[-0.024]	[-0.034]	[-0.021]	[-0.033]
Savings Ratio	-	-	0.000	0.022	-	-
			(0.016)	(0.015)		
			[0.000]	[0.002]		
Savings Dummy	-0.206**	0.016	-	-	-	-
	(0.105)	(0.106)				
	[-0.006]	[0.001]				
Safe Savings Ratio	-	-	-	-	-0.024	0.004
					(0.027)	(0.026)
					[-0.001]	[0.000]
Risky Savings Ratio	-	-	-	-	0.003	0.049*
					(0.028)	(0.026)
					[0.000]	[0.004]
Intercept	2.209	11.076***	2.159	9.787***	1.903	10.068***
	(1.469)	(1.670)	(1.488)	(1.544)	(1.512)	(1.582)
Rho	0.956	0.933	0.954	0.900	0.956	0.906
Wald Test	239.84	203.03	222.84	193.82	215.02	198.12
Degrees of freedom	11	11	11	11	12	12
p-value	0.000	0.000	0.000	0.000	0.000	0.000

*indicates statistically significant at the 10% level;

** at the 5% level;

*** at the 1% level.

## Discussion

This paper examined the links between savings as a proxy for the rate of time preference and the probability of being overweight/ obese. A priori, a negative relationship between savings and the probability of being overweight/ obese was hypothesised. Additionally, it was predicted that the negative relationship between safe savings and the probability of being overweight/ obese will be more significant than the relationship between risky savings and the probability of being overweight/ obese.

### Principal findings

Results of the regression analysis showed the action of saving (compared with not saving) is negatively and significantly related with BMI when using GMM estimation, but not when using FE estimation. According to our random-effects Probit estimations, which focus on the effect that savings has on the probability of being overweight or obese and not specifically BMI, the action of saving is not associated with the probability of being overweight or obese. Furthermore, the savings ratio was not found to be significantly related with BMI or the probability of being overweight or obese in any of the regression models.

Choosing to invest in safe savings was significantly related to a lower probability of being obese when using the random-effects Probit model only. Safe savings were not found to be significant in any of the other forms of regression estimation. The risky savings ratio was not found to be significant in any of the regression models.

Significant differences were found between the effect the safe savings ratio has on an individual’s probability of being obese, depending on their ethnicity and their level of education.

Splitting the sample by age highlights some significant differences between the older and younger subgroups. The action of saving is significantly negatively related with the probability of being overweight, for the younger subgroup only (random-effects Probit estimation). Investing in risky savings is positively associated with the probability of being overweight for the older subgroup only (random-effects Probit estimation). These results confirm our prediction that the younger subgroup would show a stronger negative relationship between savings and being overweight/obese.

It is not surprising that the different regression models show differing results, given that the different regression models use different dependent variables (dummy variables in the random-effects Probit models and continuous BMI value in FE and GMM models) and analyse the independent variables in different ways.

### Strengths and limitations of the research

To our knowledge, this is the first study to analyse the relationship between saving behaviour and being overweight/ obese in an older population where obesity prevalence levels are highest. The study also benefited from reliable BMI values rather than self-reported values as is often the case in the literature[8, 17].

A strength of the study was the use of a panel dataset for analysis. The large number of data points available helps to improve efficiency in analysis, increasing degrees of freedom[31]. Additionally, a panel dataset holds the advantage of tracking observations for individuals at different stages of their lives.

The main strengths of the research are the econometric methods used for analysis of the dataset. Using three sophisticated econometric techniques provides a helpful insight into the strength of the results. If results were found to be uniform across similar econometric methods, then we could argue there is added validity to the results. However, the results of this research differed greatly depending on the econometric method used. The FE and GMM models were both looking for the significant characteristics that affect BMI value, and the differing results across the two methods is concerning. GMM estimation can be considered superior to FE since FE estimation does not control for the possible endogeneity of the regressors. To check for endogeneity bias in random-effects Probit models and FE models, we ran the regressions again removing potentially endogenous variables: smoking, and physical activity. The results (not presented) showed no significant difference to the savings variables. The random-effects Probit model considered the effects of saving and other variables on the probability of being overweight/obese. As the FE and GMM models considered the effects of saving and other variables on the BMI value, the results of the random-effects Probit model cannot be directly compared with those from the FE and GMM models. However significant contributors to obesity would also be expected to be significant in contributing to BMI value, as the two are linked. The negative significant coefficient found for the safe savings ratio is in line with our expectations. However it must be noted that the average marginal effect (0.005) is not substantial, and smaller than the corresponding effects of other significant variables in the model (see Table 6). This paper highlights the importance for policymakers to consider the advantages and limitations in the econometric methods used before any policy recommendations are carried out off the back of regression analysis results.

Whilst the results of our comparison between saving and a short planning horizon show savings to be a reasonable proxy for the rate of time preference, there is plenty of debate as to whether savings are a good proxy. It can be argued whether savings are still a realistic proxy for time preference in an older population, given that planning ahead for the future financially may not be important in the oldest respondents. De Nardi et al.[32] showed that on average, retired individuals display asset de-accumulation as they age, especially as they approach 90 years of age. In addition, the ability to save may not just depend on one’s rate of time preference. Other factors, such as employment status, and income may restrict savings, irrespective of an individual’s rate of time preference. For this reason, all our models control for several respondents’ time-variant characteristics, as well as for unobserved time-invariant respondents’ characteristics. Moreover, our GMM specifications, that consider the saving dummy/saving ratio as endogenous take this particular point into account. Another proxy for time preference such as willpower, as examined by Zhang and Rashad[19], or survey choice questions[33, 34] may be considered more appropriate, but these are also hard to quantify. Ikeda et al.[14] use debt-holding data as a proxy for time-preference which may be a more quantifiable alternative.

Hypothesis 2 predicts investing in safe and risky savings is down to rate of time preference. Although our hypothesis is shown to be true for the random-effects Probit model for the probability of being obese (safe savings ratio), we acknowledge that choosing between investing in safe and risky savings could also be due to risk preferences rather that rate of time preference.

The savings ratio used in the study captures both active saving (what you actively save from your income) and passive saving (e.g. inheritance; interest payments), so a measure of saving that only captures active saving may have provided more convincing results. Savings can be defined in at least three different ways[35]: the difference between income and expenditure on all goods and services, either including or excluding consumer durables; or the difference in wealth between two periods[36]. The ELSA dataset does not provide sufficient information on respondents’ expenditure, which is why we define savings as the difference in wealth between two periods.

An important limitation to consider is that this research only ascertains any correlation between BMI value and household savings without addressing causality. It is unclear therefore whether significant variables, e.g. having low levels of physical activity, results in obesity, or whether obese individuals are less likely to exercise.

Other weaknesses include further shortcomings of the ELSA dataset. Item non-response was high in the data which results in the lack of continuity in the data throughout the waves. BMI value was not reported by many individuals so those observations could not be included in the analysis, despite significant differences being evident for some of the independent variables across the two groups (Table 3). However, no significant differences were found in the savings variables between the two groups which was our variable of interest. BMI value was the only measure of fatness available for all 3 waves of data. BMI value is unable to distinguish between an increase in the form of fat and an increase of lean muscle[37], and is acknowledged to be an imperfect indicator of body fat distribution in the elderly[38]. A more accurate measure of fatness, such as waist-to-hip ratio[39] would have been preferred. Furthermore, BMI value was only recorded in half of the waves, so observations were automatically halved before any analysis began.

### Comparison with similar studies

Like our study, Komlos et al.[15], Smith et al.[17] and Brown and Biosca[11] use the savings rate as a proxy for time preference. Komlos et al. consider national savings rates as a proxy and Smith et al. and Brown and Biosca[11] use savings rates at the household level, like our study. Smith et al. define savings as whether the respondent has saved or dissaved in the previous year, while Brown and Biosca look at whether respondents consider themselves a saver. Similar to our study, Smith et al. only use BMI value as a measure of fatness. However, Brown and Biosca[11] consider 3 different measures of fatness: BMI value, Waist Circumference, and percentage of body fatness. Both studies use Ordinary Least Squares regression (OLS), a commonly used method of linear regression analysis. Yet, OLS does not control for the possible endogeneity of the regressors. In contrast, our study uses GMM, which controls for endogeneity. Smith et al.[17] and Brown and Biosca also fail to track observations over time as they only analyse one wave of their dataset. Our study can be considered superior in this element, given that it maximises the potential of the panel dataset by including three waves of data, observing individuals at more than one point in time.

Smith et al. find evidence that a higher rate of time preference is positively related to body weight in men, but less in women. In line with our findings when breaking down ethnicity, they find a positive relationship specifically in black and Hispanic men and black women. Brown and Biosca find a negative relationship between saving and all three measures of fatness. Both studies highlight the limitations of using savings as a proxy for time preference.

### Future research

Future research on the links between rate of time preference and obesity in an older population is advised. The rate of time preference/obesity relationship in an older population appears less straightforward than the relationships found in previous literature concerning younger populations. Future research in this area with an older sample population should consider either using a more accurate measure of saving as a proxy for the rate of time preference, such as a measure that only captures active saving, or using other proxies that may be more appropriate for an older sample population. Furthermore, differences in the savings/obesity relationship between males and females should also be explored.

## Supporting information

S1 Table Correlation matrix between variables. (DOCX)

S2 Table Random-effects Probit model for the probability of being overweight with retired. (DOCX)

S3 Table Random-effects Probit model for the probability of being obese with retired. (DOCX)

S4 Table Fixed effects models with retired. (DOCX)

S5 Table GMM models with retired. (DOCX)

S6 Table Further regression analysis: Savings dummy (GMM model). (DOCX)

S7 Table Random-effects Probit model for the probability of being obese– Split sample by age. (DOCX)

S8 Table Fixed effects models—Split sample by age. (DOCX)

S9 Table GMM models—Split sample by age. (DOCX)

S10 Table Random-effects Probit model for the probability of being overweight—No physical activity, smoking. (DOCX)

S11 Table Random-effects Probit model for the probability of being obese—No physical activity, smoking. (DOCX)

S12 Table Fixed effects models—No physical activity, smoking. (DOCX)
