# Supplementary material for: Time to tighten the belts? Exploring the relationship between savings and obesity
Source: PLoS One. 2017 Jun 29;12(6):e0179921. doi: 10.1371/journal.pone.0179921 (PMC5491068; doi:10.1371/journal.pone.0179921)
Supplement: S1 Table — (DOCX) [file pone.0179921.s001.docx]

| *Correlation Matrix between Variables* | | | | | | | | | | | | | | | | | | |
| --- | --- | --- | --- | --- | --- | --- | --- | --- | --- | --- | --- | --- | --- | --- | --- | --- | --- | --- |
|  | BMI | Overweight Dummy | Obesity Dummy | Age | Gender | Ethnicity | Marital Status | Employment | Education | Mobility | Smoking | Income | Physical Activity | Savings Dummy | Savings Ratio | Safe savings Ratio | Risky savings Ratio | Retired |
| BMI | **1.000** |  |  |  |  |  |  |  |  |  |  |  |  |  |  |  |  |  |
| Overweight Dummy | **0.674** | **1.000** |  |  |  |  |  |  |  |  |  |  |  |  |  |  |  |  |
| Obesity Dummy | **0.796** | 0.401 | **1.000** |  |  |  |  |  |  |  |  |  |  |  |  |  |  |  |
| Age | -0.084 | -0.030 | -0.074 | **1.000** |  |  |  |  |  |  |  |  |  |  |  |  |  |  |
| Gender | -0.011 | 0.086 | -0.052 | -0.020 | **1.000** |  |  |  |  |  |  |  |  |  |  |  |  |  |
| Ethnicity | 0.022 | 0.022 | 0.016 | -0.043 | 0.025 | **1.000** |  |  |  |  |  |  |  |  |  |  |  |  |
| Marital Status | 0.027 | 0.066 | 0.000 | -0.272 | 0.196 | -0.011 | **1.000** |  |  |  |  |  |  |  |  |  |  |  |
| Employment | 0.000 | -0.012 | 0.004 | -0.499 | 0.110 | 0.004 | 0.155 | **1.000** |  |  |  |  |  |  |  |  |  |  |
| Education | -0.080 | -0.051 | -0.070 | -0.107 | 0.131 | 0.035 | 0.075 | 0.100 | **1.000** |  |  |  |  |  |  |  |  |  |
| Mobility | -0.204 | -0.124 | -0.182 | -0.252 | 0.166 | -0.011 | 0.129 | 0.251 | 0.120 | **1.000** |  |  |  |  |  |  |  |  |
| Smoking | -0.080 | -0.102 | -0.042 | -0.111 | -0.004 | -0.005 | -0.080 | 0.041 | -0.076 | -0.033 | **1.000** |  |  |  |  |  |  |  |
| Income | -0.043 | -0.008 | -0.049 | -0.212 | 0.104 | -0.031 | 0.257 | 0.300 | 0.291 | 0.183 | -0.088 | **1.000** |  |  |  |  |  |  |
| Physical Activity | -0.120 | -0.073 | -0.111 | -0.193 | 0.096 | -0.029 | 0.117 | 0.129 | 0.122 | 0.257 | -0.078 | 0.156 | **1.000** |  |  |  |  |  |
| Savings Dummy | 0.006 | -0.006 | 0.007 | -0.085 | 0.010 | 0.015 | 0.020 | 0.077 | 0.010 | -0.006 | 0.021 | 0.074 | -0.006 | **1.000** |  |  |  |  |
| Savings Ratio | 0.010 | 0.010 | 0.004 | -0.061 | 0.013 | -0.001 | 0.030 | 0.035 | 0.012 | -0.003 | 0.009 | 0.071 | 0.003 | **0.603** | **1.000** |  |  |  |
| Safe Savings Ratio | 0.006 | 0.008 | -0.007 | -0.047 | 0.001 | 0.001 | 0.021 | 0.026 | 0.000 | -0.015 | -0.012 | 0.042 | 0.002 | 0.463 | **0.691** | **1.000** |  |  |
| Risky Savings Ratio | 0.007 | 0.005 | 0.010 | -0.031 | 0.016 | -0.006 | 0.021 | 0.017 | 0.015 | 0.012 | 0.017 | 0.063 | 0.003 | 0.366 | **0.704** | -0.012 | **1.000** |  |
| Retired | -0.026 | 0.008 | -0.033 | **0.518** | -0.039 | -0.028 | -0.149 | -0.820 | -0.053 | -0.170 | -0.090 | -0.198 | -0.075 | -0.074 | -0.041 | -0.033 | -0.019 | **1.000** |
| *Correlations >0.5 are highlighted in bold.* | | | | | | | | | | | | | | | | | | |
