# Supplementary material for: Time to tighten the belts? Exploring the relationship between savings and obesity
Source: PLoS One. 2017 Jun 29;12(6):e0179921. doi: 10.1371/journal.pone.0179921 (PMC5491068; doi:10.1371/journal.pone.0179921)
Supplement: S2 Table — (DOCX) [file pone.0179921.s002.docx]

| *Random-effects Probit Model for the Probability of being Overweight with Retired* | | | |
| --- | --- | --- | --- |
| **Variable** | **Model 1: Savings Dummy** | **Model 2: Savings Ratio** | **Model 3: Safe and Risky Savings Ratios** |
| Overweight Dummy Variable | Coefficient (Standard errors in parentheses;  Average Marginal Effects in square brackets) | Coefficient (Standard errors in parentheses;  Average Marginal Effects in square brackets) | Coefficient (Standard errors in parentheses;  Average Marginal Effects in square brackets) |
| Age | -0.050***  (0.007)  [-0.002] | -0.049***  (0.007)  [-0.003] | -0.050***  (0.007)  [-0.003] |
| Gender | 1.112***  (0.109)  [0.046] | 1.091***  (0.108)  [0.057] | 1.098***  (0.109)  [0.056] |
| Ethnicity | 0.256  (0.339)  [0.011] | 0.294  (0.337)  [0.015] | 0.393  (0.342)  [0.020] |
| Marital Status | 0.491***  (0.116)  [0.020] | 0.472***  (0.115)  [0.025] | 0.467***  (0.116)  [0.024] |
| Retired | 0.185*  (0.101)  [0.008] | 0.184*  (0.100)  [0.010] | 0.202**  (0.102)  [0.010] |
| Education | -0.902***  (0.150)  [-0.038] | -0.864***  (0.148)  [-0.045] | -0.895***  (0.150)  [-0.046] |
| Mobility | -1.042***  (0.094)  [-0.044] | -1.033***  (0.093)  [-0.054] | -1.045***  (0.094)  [-0.053] |
| Smoking | -1.951***  (0.185)  [-0.081] | -1.882***  (0.178)  [-0.099] | -1.909***  (0.180)  [-0.097] |
| Income | 0.016  (0.086)  [0.001] | -0.003  (0.085)  [0.000] | 0.030  (0.086)  [0.002] |
| Physical Activity | -0.484***  (0.094)  [-0.020] | -0.493***  (0.094)  [-0.026] | -0.486***  (0.095)  [-0.025] |
| Savings Ratio | − | 0.013  (0.011)  [0.001] | − |
| Savings Dummy | -0.065  (0.071)  [-0.003] | − | − |
| Safe Savings Ratio | − | − | -0.003  (0.018)  [0.000] |
| Risky Savings Ratio | − | − | 0.024  (0.019)  [0.001] |
| Intercept | 6.099***  (0.948) | 5.969***  (0.942) | 5.808***  (0.954) |
|  |  |  |  |
| Rho | 0.944 | 0.936 | 0.938 |
|  |  |  |  |
| Wald Test | 370.19 | 378.42 | 375.57 |
| Degrees of freedom | 11 | 11 | 12 |
| p-value | 0.000 | 0.000 | 0.000 |
| **indicates statistically significant at the 10% level; ** at the 5% level; *** at the 1% level.* | | | |
