# Supplementary material for: Time to tighten the belts? Exploring the relationship between savings and obesity
Source: PLoS One. 2017 Jun 29;12(6):e0179921. doi: 10.1371/journal.pone.0179921 (PMC5491068; doi:10.1371/journal.pone.0179921)
Supplement: S3 Table — (DOCX) [file pone.0179921.s003.docx]

| *Random-effects Probit Model for the Probability of being Obese with Retired* | | | |
| --- | --- | --- | --- |
| **Variable** | **Model 1: Savings Dummy** | **Model 2: Savings Ratio** | **Model 3: Safe and Risky Savings Ratios** |
| Obese  Dummy Variable | Coefficient (Standard errors in parentheses;  Average Marginal Effects in square brackets) | Coefficient (Standard errors in parentheses;  Average Marginal Effects in square brackets) | Coefficient (Standard errors in parentheses;  Average Marginal Effects in square brackets) |
| Age | -0.080***  (0.007)  [-0.007] | -0.082***  (0.007)  [-0.008] | -0.083***  (0.007)  [-0.008] |
| Gender | -0.123  (0.109)  [-0.011] | -0.112  (0.111)  [-0.011] | -0.139  (0.112)  [-0.013] |
| Ethnicity | 0.215  (0.371)  [0.020] | 0.221  (0.375)  [0.021] | 0.292  (0.381)  [0.028] |
| Marital Status | -0.049  (0.117)  [-0.005] | -0.034  (0.119)  [-0.003] | -0.028  (0.120)  [-0.003] |
| Retired | 0.021  (0.101)  [0.002] | 0.049  (0.102)  [0.005] | 0.046  (0.104)  [0.004] |
| Education | -0.789***  (0.144)  [-0.074] | -0.800***  (0.146)  [-0.076] | -0.825***  (0.148)  [-0.078] |
| Mobility | -1.444***  (0.098)  [-0.135] | -1.464***  (0.099)  [-0.138] | -1.460***  (0.100)  [-0.139] |
| Smoking | -1.248***  (0.161)  [-0.117] | -1.292***  (0.164)  [-0.122] | -1.308***  (0.166)  [-0.124] |
| Income | -0.264***  (0.086)  [-0.025] | -0.299***  (0.088)  [-0.028] | -0.264***  (0.089)  [-0.025] |
| Physical Activity | -0.727***  (0.098)  [-0.068] | -0.740***  (0.099)  [-0.070] | -0.742***  (0.100)  [-0.070] |
| Savings Ratio | − | -0.007  (0.011)  [-0.001] | − |
| Savings Dummy | -0.055  (0.071)  [-0.005] | − | − |
| Safe Savings Ratio | − | − | -0.053***  (0.019)  [-0.005] |
| Risky Savings Ratio | − | − | 0.011  (0.019)  [0.001] |
| Intercept | 7.084***  (0.964) | 7.482***  (0.986) | 7.223***  (0.999) |
|  |  |  |  |
| Rho | 0.935 | 0.936 | 0.937 |
|  |  |  |  |
| Wald Test | 432.38 | 431.57 | 424.47 |
| Degrees of freedom | 11 | 11 | 12 |
| p-value | 0.000 | 0.000 | 0.000 |
| **indicates statistically significant at the 10% level; ** at the 5% level; *** at the 1% level.* | | | |
