# Supplementary material for: Time to tighten the belts? Exploring the relationship between savings and obesity
Source: PLoS One. 2017 Jun 29;12(6):e0179921. doi: 10.1371/journal.pone.0179921 (PMC5491068; doi:10.1371/journal.pone.0179921)
Supplement: S4 Table — (DOCX) [file pone.0179921.s004.docx]

| *Fixed Effects Models with Retired* | | | |
| --- | --- | --- | --- |
| **Variable** | **Model 1: Savings Dummy** | **Model 2: Savings Ratio** | **Model 3: Safe and Risky Savings Ratios** |
| BMI value | Coefficient (Standard errors in parentheses) | Coefficient (Standard errors in parentheses) | Coefficient (Standard errors in parentheses) |
| Age | -0.021  (0.039) | -0.014  (0.040) | -0.014  (0.040) |
| Gender | 0.000  (omitted) | 0.000  (omitted) | 0.000  (omitted) |
| Ethnicity | 0.000  (omitted) | 0.000  (omitted) | 0.000  (omitted) |
| Marital Status | 0.233  (0.164) | 0.314*  (0.167) | 0.325*  (0.170) |
| Retired | 0.038  (0.071) | 0.033  (0.072) | 0.056  (0.075) |
| Education | 0.081  (0.291) | 0.043  (0.299) | 0.067  (0.306) |
| Mobility | -0.208***  (0.070) | -0.193***  (0.070) | -0.178**  (0.072) |
| Smoking | -1.485***  (0.183) | -1.453***  (0.187) | -1.482***  (0.192) |
| Income | -0.071  (0.066) | -0.089  (0.067) | -0.074  (0.069) |
| Physical Activity | -0.048  (0.066) | -0.049  (0.067) | -0.053  (0.069) |
| Savings Ratio | − | 0.003  (0.007) | − |
| Savings Dummy | 0.001  (0.042) | − | − |
| Safe Savings Ratio | − | − | -0.002  (0.011) |
| Risky Savings Ratio | − | − | 0.006  (0.012) |
| Intercept | 30.382***  (2.844) | 29.952***  (2.885) | 29.813***  (2.924) |
|  |  |  |  |
| Rho | 0.929 | 0.929 | 0.928 |
|  |  |  |  |
| F-test  Degrees of freedom  p-value | 8.17  10  0.000 | 7.58  10  0.000 | 6.71  11  0.000 |
|  |  |  |  |
| Hausman Test  (p-value) | 351.05  0.000 | 352.75  0.000 | 346.42  0.000 |
| **indicates statistically significant at the 10% level; ** at the 5% level; *** at the 1% level.* | | | |
