# Supplementary material for: Time to tighten the belts? Exploring the relationship between savings and obesity
Source: PLoS One. 2017 Jun 29;12(6):e0179921. doi: 10.1371/journal.pone.0179921 (PMC5491068; doi:10.1371/journal.pone.0179921)
Supplement: S5 Table — (DOCX) [file pone.0179921.s005.docx]

| *GMM Models with Retired* | | | |
| --- | --- | --- | --- |
| **Variable** | **Model 1: Savings Dummy** | **Model 2: Savings Ratio** | **Model 3: Safe and Risky Savings Ratios** |
| BMI value | Coefficient (Standard errors in parentheses) | Coefficient (Standard errors in parentheses) | Coefficient (Standard errors in parentheses) |
| Age | -0.227***  (0.075) | -0.242*  (0.141) | -0.771  (5.447) |
| Gender | 0.942**  (0.385) | 0.976  (0.662) | 3.681  (30.294) |
| Ethnicity | -0.769  (1.108) | -1.487  (1.992) | -9.030  (79.418) |
| Marital Status | 0.654  (0.881) | 0.156  (1.289) | 5.486  (41.315) |
| Retired | -0.381  (0.662) | 0.110  (0.960) | -0.213  (11.850) |
| Education | 0.381  (1.521) | -0.447  (2.110) | 7.466  (63.071) |
| Mobility | -0.963  (0.680) | -1.192  (0.978) | 4.777  (68.011) |
| Smoking | -4.255  (3.748) | -8.296  (7.328) | -4.515  (72.333) |
| Income | -1.581  (3.795) | 0.803  (5.971) | -6.690  (30.406) |
| Physical Activity | -9.387*  (5.322) | -9.197  (9.076) | -57.584  (549.465) |
| Savings Ratio | − | -3.222  (3.123) | − |
| Savings Dummy | -12.602*  (6.745) | − | − |
| Safe Savings Ratio | − | − | -14.939  (87.539) |
| Risky Savings Ratio | − | − | -12.115  (150.661) |
| Intercept | 68.52229*  (39.35589) | 40.616  (55.963) | 155.413  (448.224) |
|  |  |  |  |
| F-test  Degrees of freedom  p-value | 9.90  13  0.000 | 4.90  13  0.000 | 0.32  14  0.992 |
|  |  |  |  |
| Sargan Test | 0.530 | 0.378 | Not Reported^1^ |
| **indicates statistically significant at the 10% level; ** at the 5% level; *** at the 1% level.*  *1. Sargan test is not reported as The two-step estimated covariance matrix of moments is singular.* | | | |
