# Supplementary material for: Time to tighten the belts? Exploring the relationship between savings and obesity
Source: PLoS One. 2017 Jun 29;12(6):e0179921. doi: 10.1371/journal.pone.0179921 (PMC5491068; doi:10.1371/journal.pone.0179921)
Supplement: S6 Table — (DOCX) [file pone.0179921.s006.docx]

| *Further Regression Analysis: Savings Dummy (GMM Model)* | | | |
| --- | --- | --- | --- |
|  | **Coefficient**  (Standard errors in parentheses) | | **F Statistic** |
| **Gender** | *Males* | *Females* |  |
| *Savings Dummy* | -7.503  (11.995) | -16.806  (10.385) | 0.27 |
| **Ethnicity** | *Non-white* | *White* |  |
| *Savings Dummy* | -126.570  (334.528) | -20.962  (27.310) | 0.12 |
| **Marital Status** | *Married/co-habiting* | *Not Married* |  |
| *Savings Dummy* | -8.740  (13.269) | -15.870  (11.884) | 0.11 |
| **Employment** | *Working* | *Not Working* |  |
| *Savings Dummy* | -34.398  (63.265) | -9.642  (13.188) | 0.12 |
| **Education** | *High* | *Low* |  |
| *Savings Dummy* | 2.848  (23.159) | -13.006**  (6.530) | 0.49 |
| **Mobility** | *Good* | *Bad* |  |
| *Savings Dummy* | -32.327  (67.186) | -3.594  (32.598) | 0.09 |
| **Smoking** | *Current Smoker* | *Not Current Smoker* |  |
| *Savings Dummy* | -26.923  (31.978) | -8.199  (9.744) | 0.25 |
| **Physical Activity** | *High* | *Low* |  |
| *Savings Dummy* | -5.117  (14.266) | -15.927  (9.422) | 0.38 |
| **Retired** | *Retired* | *Not Retired* |  |
| *Savings Dummy* | -11.816  (9.077) | -16.080  (21.268) | 0.03 |
| **Age** | *65 or over* | *Less than 65* |  |
| *Savings Dummy* | -7.503  (11.996) | -16.806  (10.385) | 0.27 |
| **Age** | *80 or over* | *Less than 80* |  |
| *Savings Dummy* | -7.503  (11.996) | -16.806  (10.385) | 0.27 |
| **Age** | *90 or over* | *Less than 90* |  |
| *Savings Dummy* | -7.503  (11.996) | -16.806  (10.385) | 0.27 |
| **indicates statistically significant at the 10% level; ** at the 5% level; *** at the 1% level.* | | | |
