# Supplementary material for: Time to tighten the belts? Exploring the relationship between savings and obesity
Source: PLoS One. 2017 Jun 29;12(6):e0179921. doi: 10.1371/journal.pone.0179921 (PMC5491068; doi:10.1371/journal.pone.0179921)
Supplement: S7 Table — (DOCX) [file pone.0179921.s007.docx]

| *Random-effects Probit Model for the Probability of being Obese– split sample by age* | | | | | | | | | |
| --- | --- | --- | --- | --- | --- | --- | --- | --- | --- |
| **Variable** | **Model 1: Savings Dummy** | | | **Model 2: Savings Ratio** | | | **Model 3: Safe and Risky Savings Ratios** | | |
| Obese Dummy Variable | Coefficient (Standard errors in parentheses;  Average Marginal Effects in square brackets) | | | Coefficient (Standard errors in parentheses;  Average Marginal Effects in square brackets) | | | Coefficient (Standard errors in parentheses;  Average Marginal Effects in square brackets) | | |
|  | Aged 50-69 | Aged 70+ | | Aged 50-69 | Aged 70+ | | Aged 50-69 | | Aged 70+ |
| Age | -0.023  (0.018)  [-0.001] | | -0.101***  (0.012)  [-0.011] | -0.026  (0.018)  [-0.002] | | -0.105***  (0.012)  [-0.011] | | -0.026  (0.018)  [-0.002] | -0.111***  (0.012)  [-0.011] |
| Gender | 0.195  (0.149)  [0.012] | | -0.368**  (0.149)  [-0.038] | 0.201  (0.153)  [0.014] | | -0.345**  (0.152)  [-0.036] | | 0.177  (0.151)  [0.012] | -0.400**  (0.157)  [-0.039] |
| Ethnicity | 0.157  (0.470)  [0.010] | | 0.407  (0.555)  [0.043] | 0.169  (0.485)  [0.011] | | 0.390  (0.559)  [0.041] | | 0.241  (0.478)  [0.016] | 0.469  (0.587)  [0.046] |
| Marital Status | 0.309*  (0.171)  [0.019] | | -0.328**  (0.148)  [-0.034] | 0.346**  (0.176)  [0.023] | | -0.354**  (0.151)  [-0.037] | | 0.338*  (0.173)  [0.022] | -0.347**  (0.157)  [-0.034 |
| Employment | 0.102  (0.149)  [0.006] | | -0.036  (0.294)  [-0.004] | 0.046  (0.153)  [0.003] | | -0.008  (0.298)  [-0.001] | | 0.030  (0.152)  [0.002] | 0.030  (0.307)  [0.003] |
| Education | -0.776***  (0.182)  [-0.049] | | -0.723***  (0.232)  [-0.076] | -0.789***  (0.186)  [-0.053] | | -0.734***  (0.236)  [-0.078] | | -0.817***  (0.185)  [-0.054] | -0.765***  (0.245)  [-0.075] |
| Mobility | -2.087***  (0.140)  [-0.132] | | -1.214***  (0.139)  [-0.127] | -2.110***  (0.145)  [-0.142] | | -1.236***  (0.141)  [-0.131] | | -2.085***  (0.142)  [-0.138] | -1.270***  (0.146)  [-0.124] |
| Smoking | -1.162***  (0.208)  [-0.073] | | -1.592***  (0.271)  [-0.167] | -1.250***  (0.215)  [-0.084] | | -1.580***  (0.274)  [-0.167] | | -1.267***  (0.212)  [-0.084] | -1.631***  (0.281)  [-0.160] |
| Income | -0.325***  (0.120)  [-0.021] | | -0.231*  (0.135)  [-0.024] | -0.377***  (0.123)  [-0.025] | | -0.250*  (0.138)  [-0.026] | | -0.311**  (0.123)  [-0.021] | -0.245*  (0.144)  [-0.024] |
| Physical Activity | -1.167***  (0.134)  [-0.074] | | -0.371**  (0.152)  [-0.039] | -1.180***  (0.1360  [-0.080] | | -0.375**  (0.155)  [-0.040] | | -1.198  (0.136)  [-0.079] | -0.386**  (0.160)  [-0.038] |
| Savings Ratio | - | | - | 0.000  (0.017)  [0.000] | | -0.005  (0.016)  [-0.001] | | - | - |
| Savings Dummy | -0.132  (0.107)  [-0.008] | | 0.052  (0.101)  [0.005] | - | | - | | - | - |
| Safe Savings Ratio | - | | - | - | | - | | -0.053*  (0.029)  [-0.004] | -0.068**  (0.0280  [-0.007] |
| Risky Savings Ratio | - | | - | - | | - | | 0.014  (0.029)  [0.001] | 0.035  (0.027)  [0.003] |
| Intercept | 3.187**  (1.539) | | 9.010***  (1.574) | 3.879**  (1.585) | | 9.483***  (1.617) | | 3.233**  (1.576) | 9.844***  (1.678) |
|  |  | |  |  | |  | |  |  |
| Rho | 0.968 | | 0.901 | 0.968 | | 0.903 | | 0.969 | 0.911 |
|  |  | |  |  | |  | |  |  |
| Wald Test | 365.59 | | 188.38 | 350.80 | | 185.81 | | 364.79 | 190.66 |
| Degrees of freedom | 11 | | 11 | 11 | | 11 | | 12 | 12 |
| p-value | 0.000 | | 0.000 | 0.000 | | 0.000 | | 0.000 | 0.000 |
| **indicates statistically significant at the 10% level; ** at the 5% level; *** at the 1% level.* | | | | | | | | |  |
