# Supplementary material for: Time to tighten the belts? Exploring the relationship between savings and obesity
Source: PLoS One. 2017 Jun 29;12(6):e0179921. doi: 10.1371/journal.pone.0179921 (PMC5491068; doi:10.1371/journal.pone.0179921)
Supplement: S8 Table — (DOCX) [file pone.0179921.s008.docx]

| *Fixed Effects Models – split sample by age* | | | | | | | | | |
| --- | --- | --- | --- | --- | --- | --- | --- | --- | --- |
| **Variable** | **Model 1: Savings Dummy** | | | **Model 2: Savings Ratio** | | | **Model 3: Safe and Risky Savings Ratios** | | |
| Obese Dummy Variable | Coefficient (Standard errors in parentheses) | | | Coefficient (Standard errors in parentheses) | | | Coefficient (Standard errors in parentheses) | | |
|  | Aged 50-69 | Aged 70+ | | Aged 50-69 | Aged 70+ | | Aged 50-69 | | Aged 70+ |
| Age | 0.000  (0.107) | | -0.018  (0.047) | -0.004  (0.108) | | -0.007  (0.047) | | -0.006  (0.111) | -0.007  (0.048) |
| Gender | 0.000  (omitted) | | 0.000  (omitted) | 0.000  (omitted) | | 0.000  (omitted) | | 0.000  (omitted) | 0.000  (omitted) |
| Ethnicity | 0.000  (omitted) | | 0.000  (omitted) | 0.000  (omitted) | | 0.000  (omitted) | | 0.000  (omitted) | 0.000  (omitted) |
| Marital Status | 0.344  (0.257) | | 0.125  (0.248) | 0.408  (0.261) | | 0.241  (0.253) | | 0.402  (0.263) | 0.232  (0.257) |
| Employment | -0.051  (0.1010) | | 0.342  (0.304) | -0.059  (0.103) | | 0.348  (0.311) | | -0.084  (0.105) | 0.274  (0.323) |
| Education | 0.190  (0.315) | | 0.489  (0.884) | 0.150  (0.327) | | 0.493  (0.881) | | 0.187  (0.335) | 0.465  (0.895) |
| Mobility | -0.338***  (0.099) | | -0.051  (0.121) | -0.341***  (0.100) | | -0.015  (0.122) | | -0.318***  (0.103) | 0.002  (0.128) |
| Smoking | -1.260***  (0.232) | | -1.488***  (0.397) | -1.254***  (0.240) | | -1.308***  (0.413) | | -1.302***  (0.243) | -1.292***  (0.438) |
| Income | -0.036  (0.083) | | -0.204  (0.137) | -0.052  (0.085) | | -0.235*  (0.140) | | -0.030  (0.087) | -0.217  (0.144) |
| Physical Activity | -0.159*  (0.087) | | 0.091  (0.130) | -0.172**  (0.088) | | 0.118  (0.132) | | -0.178**  (0.090) | 0.116  (0.136) |
| Savings Ratio | - | | - | 0.007  (0.009) | | -0.006  (0.011) | | - | - |
| Savings Dummy | -0.005  (0.059) | | -0.054  (0.072) | - | | - | | - | - |
| Safe Savings Ratio | - | | - | - | | - | | 0.002  (0.016) | -0.012  (0.019) |
| Risky Savings Ratio | - | | - | - | | - | | 0.014  (0.017) | -0.004  (0.020) |
| Intercept | 28.876***  (6.923) | | 31.084***  (3.866) | 29.262***  (7.032) | | 30.432***  (3.913) | | 29.159***  (7.188) | 30.280***  (3.983) |
|  |  | |  |  | |  | |  |  |
| Rho | 0.937 | | 0.911 | 0.936 | | 0.913 | | 0.936 | 0.911 |
|  |  | |  |  | |  | |  |  |
| F-test  Degrees of freedom  p-value | 5.86  10  0.000 | | 3.16  10  0.001 | 5.74  10  0.000 | | 2.98  10  0.001 | | 5.24  11  0.000 | 3.16  11  0.001 |
|  |  | |  |  | |  | |  |  |
| Hausman Test  (p-value) | 219.44  0.000 | | 137.22  0.000 | 212.39  0.000 | | 144.22  0.000 | | 209.90  0.000 | 141.38  0.000 |
| **indicates statistically significant at the 10% level; ** at the 5% level; *** at the 1% level.* | | | | | | | | |  |
