# Supplementary material for: Time to tighten the belts? Exploring the relationship between savings and obesity
Source: PLoS One. 2017 Jun 29;12(6):e0179921. doi: 10.1371/journal.pone.0179921 (PMC5491068; doi:10.1371/journal.pone.0179921)
Supplement: S9 Table — (DOCX) [file pone.0179921.s009.docx]

| *GMM Models – split sample by age* | | | | | | | | | |
| --- | --- | --- | --- | --- | --- | --- | --- | --- | --- |
| **Variable** | **Model 1: Savings Dummy** | | | **Model 2: Savings Ratio** | | | **Model 3: Safe and Risky Savings Ratios** | | |
| Obese Dummy Variable | Coefficient (Standard errors in parentheses) | | | Coefficient (Standard errors in parentheses) | | | Coefficient (Standard errors in parentheses) | | |
|  | Aged 50-69 | Aged 70+ | | Aged 50-69 | Aged 70+ | | Aged 50-69 | | Aged 70+ |
| Age | -0.139  (0.094) | | -0.131**  (0.020) | -0.058  (0.045) | | 0.158  (0.821) | | -0.006  (0.180) | -0.132  (0.746) |
| Gender | 0.825**  (0.383) | | 0.168  (0.733) | 0.562**  (0.237) | | -1.434  (0.710) | | 0.103  (1.069) | 0.063  (0.974) |
| Ethnicity | -0.396  (1.096) | | -0.154  (0.954) | 0.253  (0.645) | | 1.873  (0.737) | | -0.277  (3.572) | 0.377  (0.910) |
| Marital Status | 0.757  (1.784) | | 0.106  (0.911) | 0.057  (1.057) | | 1.287  (0.764) | | 3.001  (7.186) | -0.063  (0.933) |
| Employment | 0.702  (2.041) | | 0.529  (0.777) | -0.267  (1.155) | | -0.319  (0.945) | | 4.083  (9.162) | 0.179  (0.946) |
| Education | 0.026  (2.150) | | 0.541  (0.899) | -1.135  (1.133) | | 1.750  (0.851) | | 2.627  (8.614) | -0.507  (0.850) |
| Mobility | -1.431**  (0.718) | | -1.697***  (0.004) | -1.854***  (0.362) | | -3.758  (0.543) | | -2.030  (1.669) | -1.644  (0.764) |
| Smoking | -4.105  (3.821) | | -4.053  (0.722) | -2.216  (2.355) | | 14.537  (0.736) | | -5.631  (12.930) | -2.199  (0.795) |
| Income | -1.139  (5.789) | | -3.604  (0.747) | 0.570  (3.480) | | -7.907  (0.747) | | -12.412  (27.649) | -0.964  (0.934) |
| Physical Activity | -6.436  (4.923) | | 1.965  (0.767) | -1.669  (1.565) | | 22.902  (0.698) | | 0.457  (9.072) | 0.217  (0.996) |
| Savings Ratio | - | | - | 0.181  (0.605) | | 2.382  (0.680) | | - | - |
| Savings Dummy | -10.075  (8.462) | | 2.030  (0.792) | - | | - | | - | - |
| Safe Savings Ratio | - | | - | - | | - | | -4.950  (10.343) | -0.117  (0.979) |
| Risky Savings Ratio | - | | - | - | | - | | 4.708  (7.600) | 0.225  (0.892) |
| Intercept | 56.016  (54.954) | | 71.929  (0.496) | 28.279  (30.028) | | 87.393  (0.658) | | 146.953  (252.399) | 48.042  (0.560) |
|  |  | |  |  | |  | |  |  |
|  |  | |  |  | |  | |  |  |
| F-statistic  Degrees of freedom  P-value | 9.54  13  0.000 | | 15.01  13  0.000 | 25.63  13  0.000 | | 1.58  13  0.082 | | 1.99  14  0.015 | 16.99  14  0.000 |
|  |  | |  |  | |  | |  |  |
| Sargan Test | 0.000 | | 0.005 | 0.363 | | 0.937 | | Not reported^1^ | Not reported^1^ |
| **indicates statistically significant at the 10% level; ** at the 5% level; *** at the 1% level.*  *1. Sargan test is not reported as The two-step estimated covariance matrix of moments is singular.* | | | | | | | | |  |
