# Supplementary material for: Time to tighten the belts? Exploring the relationship between savings and obesity
Source: PLoS One. 2017 Jun 29;12(6):e0179921. doi: 10.1371/journal.pone.0179921 (PMC5491068; doi:10.1371/journal.pone.0179921)
Supplement: S10 Table — (DOCX) [file pone.0179921.s010.docx]

| *Random-effects Probit Model for the Probability of being Overweight – no physical activity, smoking* | | | |
| --- | --- | --- | --- |
| **Variable** | **Model 1: Savings Dummy** | **Model 2: Savings Ratio** | **Model 3: Safe and Risky Savings Ratios** |
| Overweight Dummy Variable | Coefficient (Standard errors in parentheses;  Average Marginal Effects in square brackets) | Coefficient (Standard errors in parentheses;  Average Marginal Effects in square brackets) | Coefficient (Standard errors in parentheses;  Average Marginal Effects in square brackets) |
| Age | -0.035***  (0.007)  [-0.001] | -0.036***  (0.007)  [-0.001] | -0.038***  (0.007)  [-0.001] |
| Gender | 1.038***  (0.106)  [0.033] | 1.042***  (0.107)  [0.032] | 1.051***  (0.108)  [0.030] |
| Ethnicity | 0.255  (0.327)  [0.008] | 0.298  (0.328)  [0.009] | 0.370  (0.331)  [0.011] |
| Marital Status | 0.578***  (0.113)  [0.018] | 0.578***  (0.114)  [0.018] | 0.571***  (0.115)  [0.016] |
| Employment | -0.253**  (0.114)  [-0.008] | -0.263**  (0.115)  [-0.008] | -0.299**  (0.117)  [-0.008] |
| Education | -0.793***  (0.146)  [-0.025] | -0.783***  (0.148)  [-0.024] | -0.809***  (0.150)  [-0.023] |
| Mobility | -1.008***  (0.091)  [-0.032] | -1.011***  (0.092)  [-0.031] | -1.019***  (0.093)  [-0.029] |
| Income | 0.076  (0.085)  [0.002] | 0.061  (0.086)  [0.002] | 0.100  (0.088)  [0.003] |
| Savings Ratio | - | 0.012  (0.011)  [0.000] | - |
| Savings Dummy | -0.053  (0.070)  [-0.002] | - | - |
| Safe Savings Ratio | - | - | -0.004  (0.018)  [0.000] |
| Risky Savings Ratio | - | - | 0.024  (0.019)  [0.001] |
| Intercept | 4.242***  (0.916) | 4.394***  (0.929) | 4.233***  (0.942) |
|  |  |  |  |
| Rho | 0.944 | 0.944 | 0.946 |
|  |  |  |  |
| Wald Test | 258.58 | 257.32 | 256.06 |
| Degrees of freedom | 9 | 9 | 10 |
| p-value | 0.000 | 0.000 | 0.000 |
| **indicates statistically significant at the 10% level; ** at the 5% level; *** at the 1% level.* | | | |
