# Supplementary material for: Time to tighten the belts? Exploring the relationship between savings and obesity
Source: PLoS One. 2017 Jun 29;12(6):e0179921. doi: 10.1371/journal.pone.0179921 (PMC5491068; doi:10.1371/journal.pone.0179921)
Supplement: S11 Table — (DOCX) [file pone.0179921.s011.docx]

| *Random-effects Probit Model for the Probability of being Obese – no physical activity, smoking* | | | |
| --- | --- | --- | --- |
| **Variable** | **Model 1: Savings Dummy** | **Model 2: Savings Ratio** | **Model 3: Safe and Risky Savings Ratios** |
| Overweight Dummy Variable | Coefficient (Standard errors in parentheses;  Average Marginal Effects in square brackets) | Coefficient (Standard errors in parentheses;  Average Marginal Effects in square brackets) | Coefficient (Standard errors in parentheses;  Average Marginal Effects in square brackets) |
| Age | -0.069***  (0.007)  [-0.006] | -0.070***  (0.007)  [-0.006] | -0.071***  (0.007)  [-0.006] |
| Gender | -0.167  (0.109)  [-0.014] | -0.154  (0.110)  [-0.013] | -0.177  (0.111)  [-0.015] |
| Ethnicity | 0.323  (0.367)  [0.027] | 0.329  (0.368)  [0.028] | 0.387  (0.375)  [0.033] |
| Marital Status | 0.008  (0.115)  [0.001] | 0.024  (0.117)  [0.002] | 0.030  (0.118)  [0.003] |
| Employment | -0.106  (0.117)  [-0.009] | -0.140  (0.118)  [-0.012] | -0.149  (0.120)  [-0.013] |
| Education | -0.785***  (0.142)  [-0.065] | -0.793***  (0.144)  [-0.067] | -0.815***  (0.146)  [-0.069] |
| Mobility | -1.494***  (0.097)  [-0.123] | -1.503***  (0.098)  [-0.128] | -1.503***  (0.099)  [-0.127] |
| Income | -0.232***  (0.086)  [-0.019] | -0.261***  (0.088)  [-0.022] | -0.228**  (0.089)  [-0.019] |
| Savings Ratio | - | -0.007  (0.011) | - |
| Savings Dummy | -0.043  (0.070)  [-0.004] | [-0.001] | - |
| Safe Savings Ratio |  | - | -0.049***  (0.019)  [-0.004] |
| Risky Savings Ratio | - | - | 0.010  (0.019)  [0.001] |
| Intercept | 5.647***  (0.942) | 6.016***  (0.959) | 5.777***  (0.972) |
|  |  |  |  |
| Rho | 0.939 | 0.939 | 0.940 |
|  |  |  |  |
| Wald Test | 351.24 | 352.34 | 347.38 |
| Degrees of freedom | 9 | 9 | 10 |
| p-value | 0.000 | 0.000 | 0.000 |
| **indicates statistically significant at the 10% level; ** at the 5% level; *** at the 1% level.* | | | |
