# Supplementary material for: Time to tighten the belts? Exploring the relationship between savings and obesity
Source: PLoS One. 2017 Jun 29;12(6):e0179921. doi: 10.1371/journal.pone.0179921 (PMC5491068; doi:10.1371/journal.pone.0179921)
Supplement: S12 Table — (DOCX) [file pone.0179921.s012.docx]

| *Fixed Effects Models – no physical activity, smoking* | | | |
| --- | --- | --- | --- |
| **Variable** | **Model 1: Savings Dummy** | **Model 2: Savings Ratio** | **Model 3: Safe and Risky Savings Ratios** |
| BMI value | Coefficient (Standard errors in parentheses) | Coefficient (Standard errors in parentheses) | Coefficient (Standard errors in parentheses) |
| Age | -0.023  (0.040) | -0.015  (0.040) | -0.015  (0.041) |
| Gender | 0.000  (omitted) | 0.000  (omitted) | 0.000  (omitted) |
| Ethnicity | -0.967  (1.070) | -0.980  (1.070) | -1.006  (1.080) |
| Marital Status | 0.223  (0.165) | 0.317*  (0.167) | 0.325*  (0.170) |
| Employment | -0.127  (0.089) | -0.133  (0.091) | -0.165*  (0.093) |
| Education | 0.092  (0.294) | 0.055  (0.301) | 0.078  (0.308) |
| Mobility | -0.220***  (0.070) | -0.206***  (0.071) | -0.191***  (0.073) |
| Income | -0.048  (0.068) | -0.058  (0.069) | -0.044  (0.071) |
| Savings Ratio | - | 0.002  (0.007) | - |
| Savings Dummy | 0.017  (0.042) | - | - |
| Safe Savings Ratio | - | - | -0.002  (0.011) |
| Risky Savings Ratio | - | - | 0.006  (0.012) |
| Intercept | 30.123***  (2.873) | 29.678***  (2.913) | 29.574***  (2.950) |
|  |  |  |  |
| Rho | 0.928 | 0.928 | 0.927 |
|  |  |  |  |
| F-test  Degrees of freedom  p-value | 1.85  9  0.055 | 1.92  9  0.045 | 1.67  10  0.081 |
|  |  |  |  |
| Hausman Test  (p-value) | 272.51  0.000 | 275.18  0.000 | 271.50  0.000 |
| **indicates statistically significant at the 10% level; ** at the 5% level; *** at the 1% level.* | | | |
